# Cardiovascular sequelae of Long COVID: immune dysregulation inflammation as central drivers

**DOI:** 10.3389/fimmu.2026.1815269

**Published:** 2026-06-17

**Authors:** Anhui Liu, Hanbing Chen, Qingxiang Liu, Mahmoud Al-Azab, Fahmi Shaher, Jiang Li, Tao Liu, Min Yang

**Affiliations:** 1 Wuxi School of Medicine, Jiangnan University, Wuxi, China; 2 Department of Cardiac Vascular Surgery, Affiliated Hospital of Jiangnan University, Wuxi, China; 3 Department of Cardiac Surgery, Peking University People's Hospital, Peking University, Beijing, China; 4 School of Medicine, Southeast University, Nanjing, China; 5 Department of Emergency Medicine, The First Affiliated Hospital with Nanjing Medical University, Nanjing, China; 6 School of Basic Medical Sciences, Guangzhou Women and Children’s Medical Centre, Guangzhou Medical University, Guangzhou, China; 7 Department of Medical Microbiology, Faculty of Medicine, University of Science and Technology, Aden, Yemen; 8 Department of Basic Sciences, Faculty of Medicine, University of Science and Technology, Aden, Yemen; 9 Clinical Big Data Research Center, The Seventh Affiliated Hospital of Sun Yat-sen University, Shenzhen, China; 10 State Key Laboratory of Respiratory Disease, National Clinical Research Center for Respiratory Disease, National Center for Respiratory Medicine, Joint International Research Laboratory of Respiratory Health, Guangdong Basic Research Center of Excellence for Respiratory Medicine, Department of Allergy and Clinical Immunology, Guangzhou Institute of Respiratory Health, The First Affiliated Hospital of Guangzhou Medical University, Guangzhou, China

**Keywords:** adaptive immune response, cardiovascular sequelae, immune dysregulation, innate immune response, Long COVID

## Abstract

Long coronavirus disease 2019 (Long COVID-19), also referred to as post-acute sequelae of severe acute respiratory syndrome coronavirus 2 (SARS-CoV-2) infection, has emerged as a major global health challenge. Common manifestations include fatigue, dyspnea, cognitive dysfunction, and exercise intolerance. Beyond these systemic manifestations, the enduring cardiovascular manifestations are increasingly identified as core characteristics of Long COVID-19 syndromes secondary to SARS-CoV-2 infection, encompassing myocarditis, ischemic and non-ischemic heart disease, arrhythmias, heart failure, and thrombotic events. Accumulating evidence suggests that immune dysregulation and persistent inflammation are central drivers of cardiovascular injury in Long COVID. Persistent activation of innate and adaptive immune pathways fosters endothelial injury, thrombo-inflammation, and adverse myocardial remodeling. In this review, we focus on current clinical and experimental evidence to delineate the immune-mediated mechanisms underlying cardiovascular sequelae in Long COVID and explore potential therapeutic strategies targeting persistent inflammation and immune dysregulation.

## Introduction

Long COVID, as defined by the World Health Organization (WHO), refers to symptoms that persist beyond 3 months from the onset of SARS-CoV-2 infection, last for at least 2 months, and cannot be attributed to alternative diagnosis (1). It has emerged as a critical public health and socioeconomic concern. However, the prevalence of Long COVID varies widely and is challenging to estimate accurately, which may be attributable to the high sensitivity of prevalence estimates to differences in definitions of Long COVID, study populations, inclusion criteria, statistical methodologies, and periods of COVID-19 pandemic (2). An interdisciplinary review suggested that the prevalence of Long COVID varies by disease severity and vaccination status, ranging from 50-85% in unvaccinated hospitalized patients to 10-35% in unvaccinated non-hospitalized individuals, and further declining to 8-12% among vaccinated populations (3). In contrast to the previously reported high incidence of Long COVID, data from the Office for National Statistics reported that 3-11.7% of COVID-19 patients experienced persistent symptoms consistent with Long COVID 12 weeks following infection (4). Clinically, Long COVID represents a heterogeneous multisystem disorder affecting the respiratory, cardiovascular, neurological, gastrointestinal, and musculoskeletal systems, and is characterized by diverse symptoms including fatigue, dyspnea, cardiovascular abnormalities, cognitive impairment, sleep disturbances, post-traumatic stress-related symptoms, myalgia, impaired concentration, and headache (5). Among the affected organ systems, the cardiovascular system has attracted particular attention due to the frequency, severity, and potential irreversibility of its involvement. A large cohort study using US Veterans Affairs databases (n=153, 760 COVID-19 cases with over 5 million controls) demonstrated that beyond 30 days after infection, COVID-19 was associated with a significantly increased 1-year risk of incident cardiovascular diseases, including myocarditis, arrhythmias, ischemic and non-ischemic heart disease, heart failure, and thromboembolic events (6).

The molecular mechanisms underlying the development of Long COVID, particularly in those cardiovascular diseases, are highly complex. A cohort study indicates that immune dysregulation in Long COVID is not uniform and may manifest as two immunological types, including immune activation, leading to persistent inflammation, and impaired immune function, potentially promoting viral persistence and contributing to ongoing tissue injury and prolonged symptoms (7). Emerging studies showed that sustained immune dysregulation may act as contributing drivers of cardiovascular sequelae in Long COVID (8). A multicenter cross-sectional study of 275 participants reported that Long COVID patients exhibited significant immunological differences, including significant changes in circulating immune cell populations, compared with healthy controls and convalescent COVID-19 patients (9). However, findings of immune dysfunction across studies remain inconsistent. Some studies suggested that immune imbalance in individuals with Long COVID tends to improve over the course of disease. A longitudinal cohort study demonstrated that 62% of Long COVID patients exhibited a gradual normalization of biomarkers related to immune dysregulation at 2 years after COVID-19 infection (10). A recent longitudinal study with a 40-month follow-up reported that persistent immune dysregulation may not be present in all Long COVID patients, with no significant differences in antibody and T cell response among Long COVID patients, recovered individuals, and healthy controls (11). Differences in study design, population selection, follow-up duration, and outcome measures contribute to heterogeneity in findings on immune dysregulation in Long COVID. This heterogeneity has important implications for understanding the mechanisms of cardiovascular complications and suggests that immune-mediated injury may not occur uniformly across all patients but may instead depend on individual immunological characteristics and disease trajectories.

Immune dysregulation, including both innate and adaptive immune response, is increasingly recognized as a fundamental mechanism underlying the development and progression of diverse diseases (12–17). Innate immune imbalance, characterized by aberrant pattern recognition receptors.

(PRRs) signaling, sustained inflammasome activation, excessive neutrophil extracellular trap formation, and complement dysregulation, drives persistent inflammatory activation (18–27). In parallel, adaptive immune dysregulation, including persistent antigen stimulation, T-cell exhaustion, T Cell subset imbalance, and dysregulated B-cell responses, further promotes chronic inflammation, thereby contributing to multisystem pathology (28–30). This review focuses on discussing the immune-mediated pathways that contribute to cardiovascular complications in Long COVID. By integrating current clinical and mechanistic evidence, we aim to provide a conceptual framework for understanding immune-driven cardiovascular injury in Long COVID.

## Cardiovascular manifestations of Long COVID

**Figure 1:**
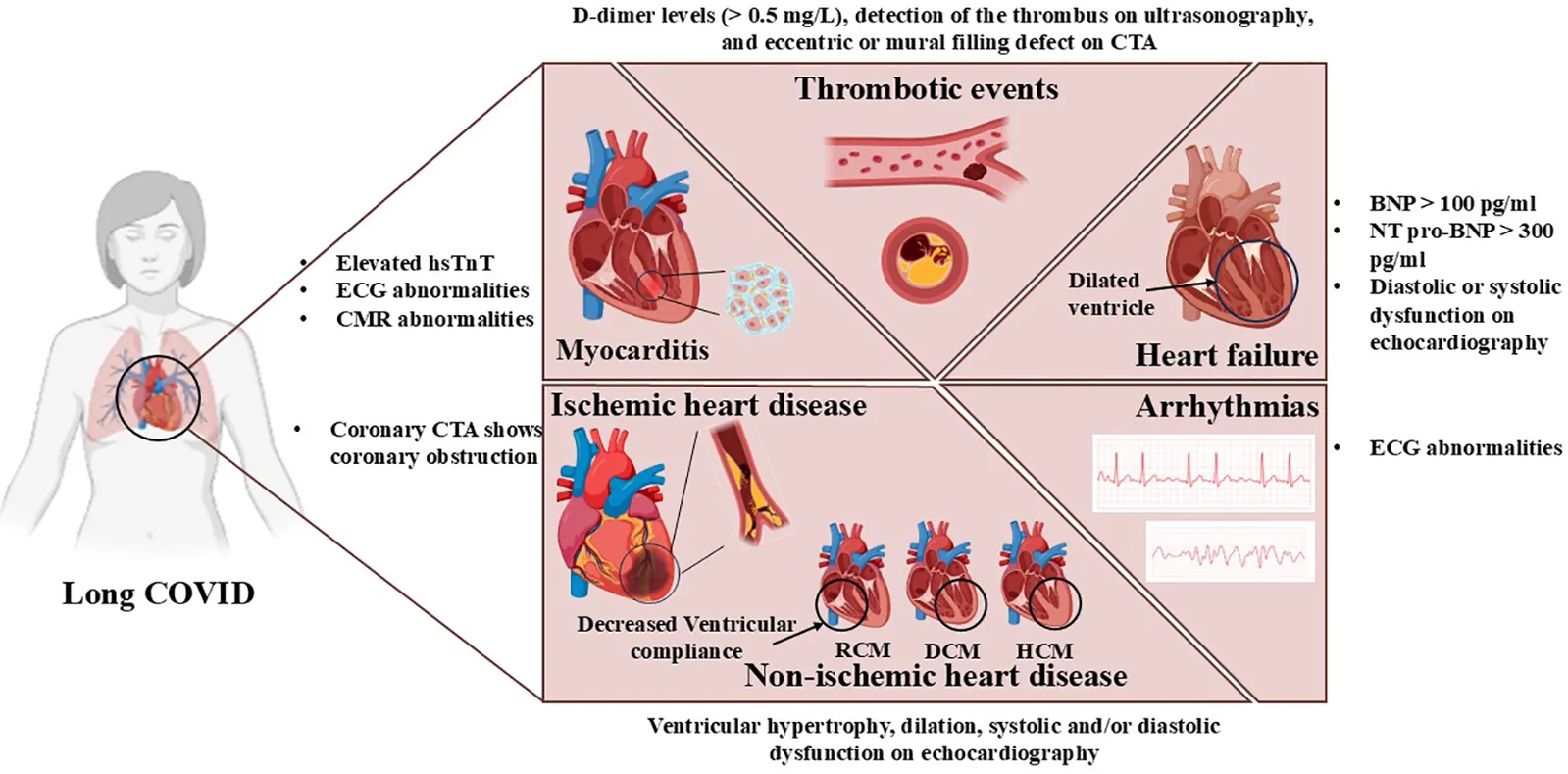
The cardiovascular manifestations of Long COVID. Long COVID patients commonly manifest cardiovascular involvement, including myocarditis, ischemic and non-ischemic heart disease, arrhythmias, heart failure, and thrombotic events. Myocarditis is diagnosed by elevated high sensitivity troponin T (hsTnT) and abnormalities in electrocardiogram (ECG) and cardiac magnetic resonance imaging (CMR). Ischemic heart disease presents with coronary obstruction on computed tomographic angiography (CTA). Non-ischemic heart disease includes restrictive cardiomyopathy (RCM), dilated cardiomyopathy (DCM), and hypertrophic cardiomyopathy (HCM), which presents with ventricular hypertrophy, dilation, systolic and/or diastolic dysfunction on echocardiography. Arrhythmias are typically diagnosed by ECG abnormalities. The diagnosis of heart failure depends on laboratory and imaging abnormalities, including brain natriuretic peptide (BNP) > 100 pg/ml, N-terminal pro-BNP (NT pro-BNP) >300 pg/ml, and diastolic or systolic dysfunction on echocardiography. Thrombotic events are commonly diagnosed by elevated D-dimer levels (> 0.5 mg/L), detection of the thrombus on ultrasonography, and eccentric or mural filling defect on CTA.

Cardiovascular sequelae of Long COVID impose a substantial burden on both individuals and healthcare systems. Accumulating evidence indicates that patients with Long COVID are at high risk of developing long-term cardiovascular complications, including myocarditis, ischemic and non-ischemic heart disease, arrhythmias, heart failure (HF), and thrombotic events (6, 31–33) (**Figure 1**).

### Myocarditis

Myocarditis is defined as myocardial inflammation characterized by multifocal inflammatory infiltrate and associated with cardiac injury, which may result in cardiac dysfunction (8, 34). The symptoms of myocarditis range from asymptomatic presentation to chest pain, dyspnea, fatigue, and palpitations, and in severe cases may lead to syncope, cardiogenic shock, and death (35, 36). According to the duration of symptoms, myocarditis can be classified into fulminant (< 2 weeks), acute (< 4 weeks), subacute (4 weeks to 3 months), and chronic (> 3 months) forms (37). The diagnosis typically relies on laboratory and imaging abnormalities, including elevated serum high-sensitivity troponin T (hsTnT), defined as exceeding the 99th percentile upper reference limit, and abnormalities in electrocardiogram (ECG) such as sinus tachycardia, ST-segment and T-wave abnormalities, as well as cardiac magnetic resonance imaging (CMR) findings such as late gadolinium enhancement (LGE), which is suggestive of myocardial injury (32, 35, 38, 39).

In the acute phase of SARS-CoV-2 infection, myocardial involvement has been increasingly recognized, ranging from acute myocarditis to fulminant myocarditis (40). In a retrospective study of 21 COVID-19 patients, 3 (14%) patients diagnosed with myocarditis presented with elevated troponin levels accompanied by ECG abnormalities, such as atrial fibrillation or new ST-segment depression (34). Another autopsy study of 15 COVID-19 patients reported that 26.7% exhibited inflammatory infiltrates consistent with myocarditis (41). Beyond the acute phase, emerging evidence indicates that myocarditis is a recognized complication of Long COVID (42). In a prospective cohort study of 100 patients recovering from COVID-19, 71% showed elevated hsTnT levels (> 3 pg/mL), 78% demonstrated cardiac involvement on CMR, and 60% had ongoing myocardial inflammation (43). However, the reported high prevalence of myocarditis in this study has been widely debated because non-standard CMR thresholds were applied as diagnostic criteria in patients without clinical suspicion of myocarditis (44). In contrast, another cross-sectional study of 139 healthcare workers with Long COVID reported that 25.2% of participants met the diagnostic criteria for myocarditis, with characteristic abnormalities in ECG or CMR and cardiopulmonary symptoms such as chest pain, dyspnea or palpitations (45). This incidence of myocarditis appears to be comparable to reported rates during the acute phase of COVID-19. Overall, the reported prevalence of myocarditis associated with Long COVID varies substantially across studies, which may be attributable to differences in study design, patient populations, and diagnostic criteria. While the precise risk of myocarditis remains uncertain, the individuals recovering from COVID-19 who present with the aforementioned cardiopulmonary symptoms may warrant further clinical evaluation and close surveillance.

### Ischemic and non-ischemic heart disease

According to whether cardiac injury is caused by the coronary artery stenosis, cardiac diseases are classified into ischemic heart disease (IHD) and non-ischemic heart disease. IHD includes angina and acute coronary syndromes (ACS), such as ST-segment elevation myocardial infarction (STEMI) and non-ST-segment elevation myocardial infarction (NSTEMI), which typically presents with symptoms such as chest pain, sweating, and breathlessness, with the evidence of coronary obstruction on coronary computed tomographic angiography (CTA) (46). Non-ischemic heart disease, including dilated cardiomyopathy (DCM), restrictive cardiomyopathy (RCM), and hypertrophic cardiomyopathy (HCM), is characterized by structural and/or functional abnormalities, presenting with ventricular hypertrophy, dilation, systolic and/or diastolic dysfunction on echocardiography (47).

Accumulating evidence suggests that the risk of IHD is increased during the acute phase of SARS-CoV-2 infection. Supporting this notion, a self-controlled case series study reported that COVID-19 patients had a 2.89-fold increased risk of IHD during the first week following infection (95% *CI*: 1.51-5.55) (48). Notably, hospitalized COVID-19 patients with STEMI had a poor prognosis, with reported mortality rates of 12-72% (49). However, reports on new onset non-ischemic heart disease are comparatively limited. Available studies indicate that COVID-19 may exacerbate pre-existing cardiomyopathy. A multicenter study of COVID-19 patients with pre-existing DCM and HCM showed that the patients experienced a significant deterioration in physical health compared with those without non-ischemic heart disease (31.3% vs 13.2%, *P*=0.004) (50). Beyond the acute phase of COVID-19, Long COVID patients may also experience IHD or non-ischemic heart disease. During the convalescent phase of COVID-19, Long COVID patients were also at increased risk of IHD (*HR*: 1.81, 95% *CI*: 1.77-1.84), including ACS and angina, with the increased risk persisting for up to 18 months, according to a retrospective cohort study from Japan and Korea (51). Similarly, another retrospective study reported that Long COVID patients one year after onset had a significantly increased risk of non-ischemic cardiomyopathy (HR: 1.62, 95% CI: 1.52-1.73) (6). Overall, current evidence suggests that Long COVID patients are at potential risk of developing both ischemic and non-ischemic heart diseases, regardless of pre-existing cardiovascular conditions.

### Arrhythmias

Arrhythmias, characterized by abnormalities in cardiac electrical activity, are classified into tachyarrhythmias (heart rates > 100 beats/min) and bradyarrhythmias (< 60 beats/min), both of which may present with dizziness, lightheadedness, fatigue and dyspnea at rest or during exertion secondary to decreased blood pressure and cerebral hypoperfusion (52, 53). Tachyarrhythmias include atrial fibrillation (AF), ventricular fibrillation (VF), supraventricular tachycardia (SVT), and ventricular tachycardia (VT), and bradyarrhythmias include sinus bradycardia and atrioventricular block (AVB) (52).

During the acute phase of SARS-CoV-2 infection, arrhythmias are recognized as common complication, with an incidence of approximately 7.4% to 16.7% (54, 55). A worldwide cross-sectional study of 1197 hospitalized COVID-19 patients by the Heart Rhythm Society demonstrated that AF was the most common tachyarrhythmias (21%), while severe sinus bradycardia (8%) and complete AVB (8%) were the predominant forms of bradyarrhythmias (56). Similarly, Long COVID patients also remain at high risk of arrhythmias (57). A meta-analysis of 14 retrospective cohort studies demonstrated that Long COVID patients had a 74% increased risk of arrhythmias compared to non-COVID-19 patients (*HR*: 1.74, 95% *CI*: 1.39-2.10, *I^2^* = 99.65%) (58). Consistent with this study, another follow-up study of a million patients recovering from COVID-19 indicated that the risk of arrhythmias related to Long COVID increased significantly (*HR*: 6.44, 95% *CI*: 4.17-9.96) (59). In addition, postural orthostatic tachycardia syndrome (POTS) is a special type of tachyarrhythmias associated with dysautonomia, presenting with dizziness, breathlessness, sweating, and bloating, which may be triggered by standing or minimal exertion (60). Reports have indicated that COVID-19 can induce POTS during the acute phase. A retrospective study of 27 COVID-19 patients reported that nearly 22% of them met the criteria for POTS (61). Similarly, POTS has also been reported in Long COVID patients. Current studies have shown that 2-14% of Long COVID patients are diagnosed with POTS, while 9-61% exhibit POTS-like dysautonomia symptoms that do not meet the diagnostic criteria (60, 62). The wide range in reported prevalence may be attributed to differences in examined populations, the broad spectrum of dysautonomia-related symptoms, and imprecise diagnostic criteria for POTS. Given these limitations, improved strategies for early identification and risk stratification are needed. Thus, Continuous arrhythmia monitoring, such as the use of wearable smart technologies, may facilitate early detection and risk assessment of individuals at high risk (63).

### Heart failure

Heart failure (HF) is a clinical syndrome resulting from structural and/or functional cardiac abnormalities, manifesting as elevated cardiac biomarkers, such as natriuretic peptide levels (NPs) and hsTnT, together with abnormal findings on echocardiography (64, 65). These laboratory and imaging abnormalities are typically characterized by elevated brain natriuretic peptide (BNP) levels (> 100 pg/ml) or N-terminal pro-BNP (NT-pro BNP) levels (> 300 pg/ml), as well as echocardiographic findings of adverse diastolic or systolic dysfunction (66).

Accumulating studies provide evidence that the burden of HF in COVID-19 patients is significantly increased (*HR*: 3.94, 95%*CI*: 2.97-4.80) (67), with approximately 23% of patients developing HF, of which 52% resulted in mortality (68). Similarly, Long COVID is closely associated with incidence of HF. Supporting this notion, a meta-analysis of Long COVID patients with a mean follow-up of 9.2 months after SARS-CoV-2 infection demonstrated that Long COVID patients exhibited a significantly elevated long-term risk of heart failure (*HR*: 1.90, 95% *CI*: 1.54-3.24, *P* < 0.0001, *I^2^* = 96.5%) (69). Consistent with these findings, a follow-up study including 81 COVID-19 patients discharged for 6 weeks found that 62% of patients exhibited cardiac dysfunction, with most classified as New York Heart Association (NYHA) class II-III (70). Another observational, multicenter, prospective study of 145 COVID-19 patients demonstrated that 60% and 55% of patients exhibited diastolic dysfunction on transthoracic echocardiography at 60 and 100 days after COVID-19 infection, respectively, with 23% elevated NT-pro BNP 100 days after infection (71). Furthermore, Long COVID may exacerbate the cardiovascular burden in patients with pre-existing coronary artery conditions, thus contributing to adverse cardiovascular outcomes (72). Individuals with pre-existing cardiovascular comorbidities who developed HF 4 years after SARS-CoV-2 infection showed a markedly elevated incidence compared to those without SARS-CoV-2 infection (*HR*: 1.58, 95% *CI*: 1.38-1.8, *P* < 0.05) (73). In summary, these findings demonstrate that Long COVID patients are at high risk of HF, which exerts a burden on public health and requires long-term clinical surveillance.

### Thrombosis

Thrombosis is defined as the formation of a hemostatic plug or blood clot within a vessel, commonly accompanied by coagulopathies, which potentially leads to partial or complete vascular obstruction (74, 75). Furthermore, some studies describe microclots as fibrinaloid microclots with amyloid properties (76, 77). In contrast to this notion, other studies suggest that the term of microclots, commonly described as a mixture of fibrin mesh and platelets, may not accurately characterize coagulopathies in Long COVID. Instead, these particles are proposed to consist of amyloid and fibrinogen (78, 79). Although the hypothesis of microclots remains controversial, accumulating evidence consistently indicates that patients with Long COVID are at an increased risk of thrombotic events. Depending on the location of the thrombus, thrombosis may lead to venous thrombosis, arterial thrombosis, myocardial infarction, pulmonary embolism, or stroke. Clinically, thrombosis is characterized by elevated D-dimer levels (> 0.5 mg/L), which are associated with an increased risk of thrombotic events, as well as imaging findings such as the detection of the thrombus on ultrasonography, and eccentric or mural filling defect on CTA (74, 80).

During the acute phase of SARS-CoV-2 infection, COVID-19 patients are at high risk of thrombotic events, accompanied with elevated biomarkers levels such as D-dimer. Supporting this notion, a multicenter and prospective cohort study of 295 patients demonstrated that the incidence of thrombotic events was significantly higher in patients with COVID-19 (n=150) than in non-COVID-19 patients (n=145) (11.7% vs. 2.1%, *P* < 0.008), with elevated D-dimer levels observed in more than 95% of patients (81). Similarly, a multicenter and cross-sectional study involving 384 COVID-19 patients 4-6 weeks after discharge reported that 30.1% had persistently elevated D-dimer levels (82). Beyond the acute phase of COVID-19, the risk of thrombotic events increases significantly in Long COVID patients. A retrospective cohort study from the TriNetX US Collaborative Network demonstrated that Long COVID patients were at increased risk of thromboembolic disorders during 12 months of follow-up (*HR*: 2.648, 95% *CI*: 2.443-2.870) (83). In addition, increasing evidence demonstrates that venous thrombosis occurs more frequently than arterial thrombosis. A multicenter cohort study of 48 million people from England and Wales demonstrated a persistently elevated risk of venous thrombosis compared with arterial thrombosis 27-49 weeks after COVID-19 diagnosis (*HR*: 1.80, 95% *CI*: 1.50-2.17 vs. *HR*: 1.34, 95% *CI*: 1.21-1.48) (84). In conclusion, Long COVID is closely associated with thrombosis, imposing a substantial burden on healthcare systems. Therefore, further surveillance of D-dimer and early prophylactic anticoagulation are essential for patients at high risk of thrombotic events.

## The impact of vaccination on cardiovascular sequelae of Long COVID

Considering the substantial healthcare burden of Long COVID, it remains essential to clarify whether vaccination reduces the risk of Long COVID and related cardiovascular sequelae. Emerging studies report a declining trend in incidence of Long COVID and related complications following vaccination. According to a community-based cohort study, the incidence of persistent symptoms three months after SARS-CoV-2 infection was lower in vaccinated individuals than in unvaccinated individuals (9.5% vs 14.6%, *HR*=0.59, 95% *CI*: 0.50-0.69) (85). Similarly, a 79% reduction in referrals of patients with Long COVID symptoms lasting at least five months was observed at Cambridge University Teaching Hospital following the second dose of vaccination in the United Kingdom (86). A cross-sectional study in Israel reported that patients who developed SARS-CoV-2 infection after receiving two doses of BNT162b2 mRNA vaccine were less likely than unvaccinated individuals to report chronic symptoms, such as fatigue, headache, limb weakness, and persistent muscle pain, during the follow-up period (87). Consistent with these findings, a large cohort study reported that people with SARS-CoV-2 infection after vaccination (n=33, 940) exhibited lower risks of death (*HR*=0.66, 95% *CI*: 0.58-0.74) and Long COVID (*HR*=0.85, 95% *CI*: 0.82-0.89), including cardiovascular, coagulation and hematologic disorders, during a six-month follow-up period, compared with unvaccinated individuals with SARS-CoV-2 infection (n=113, 474) (88). Given differences in study populations, follow-up duration, vaccine types, SARS-CoV-2 variants, and study methodologies, findings across studies are heterogeneous, but they generally suggest that vaccination is associated with a reduced risk of Long COVID and related cardiovascular sequelae.

## Immune mechanisms of cardiovascular injury in Long COVID

Persistent immune activation following SARS-CoV-2 infection has emerged as a hallmark of Long COVID. Accumulating evidence indicates that continuous viral antigens stimulation, aberrant innate and adaptive immune responses contribute to the chronic inflammatory state observed in Long COVID (89). Supporting this notion, integrative immunological, virological, transcriptomic, and proteomic analyses of a longitudinal cohort (n=142) revealed that patients with Long COVID exhibited persistent immune activation and heightened proinflammatory signatures for more than 180 days after initial infection, in contrast to convalescent controls (90).

## Innate immune dysregulation in Long COVID

### PRRs activation and endothelial injury

Innate immune response serves as the host’s first line of defense against pathogens and damage-associated stimuli. Recognition of these signals by PRRs triggers the activation of downstream cascades (91). Persistent activated PRRs have emerged as one of the central features linking SARS-CoV-2 infection to chronic immune dys-regulation and cardiovascular complications observed in Long COVID. PRRs, including Toll-like receptors (TLRs), RIG-I-like receptors (RLRs), and NOD-like receptors (NLRs), are critical sensors of pathogen-associated molecular patterns (PAMPs) and damage-associated molecular patterns (DAMP). During acute SARS-CoV-2 infection, viral genomic RNAs are recognized by TLR3, TLR7, and RIG-I/MDA5, which initiate downstream nuclear factor kappa-light-chain-enhancer of activated B cells (NF-kB) and interferon regulatory factor (IRF) signaling pathways. These cascades rapidly induce transcription of pro-inflammatory cytokines such as TNF-a, IL-6, and IL-1b, as well as type I and III interferons that establish an antiviral state (92–94). Although this early innate immune activation is essential for restricting viral replication, it may simultaneously promote systemic inflammation and endothelial dysfunction (95). Importantly, accumulating evidence shows that PRR signaling does not fully resolve following viral clearance, but instead persists for weeks to months, contributing to chronic immune activation in Long COVID patients (96).

The persistence of PRRs activation can be attributed to several factors. First, residual viral RNA fragments and defective genomes have been detected in multiple tissues long after acute infection, including cardiac tissue, vascular endothelium, gut epithelium, and lymphoid compartments (97–99). These RNA remnants, though replication-incompetent, retain sufficient structural motifs to stimulate RNA sensors such as TLR7 and RIG-I, perpetuating low-level interferon and cytokine signaling. Second, tissue damage during acute infection generates abundant DAMPs, including extracellular mitochondrial DNA, oxidized phospholipids, high mobility group box 1 protein (HMGB1), and extracellular histones. These endogenous ligands serve as potent activators of PRRs, particularly TLR2, TLR4, and cytosolic DNA sensors such as cGAS-STING (100–102). Third, emerging evidence suggests that SARS-CoV-2 infection induces epigenetic reprogramming of innate immune cells, a phenomenon termed “trained immunity, ” in which chromatin accessibility and metabolic rewiring prime monocytes and macrophages for exaggerated inflammatory responses upon subsequent stimulation (103–105). Furthermore, transcriptomic analyses have identified prolonged activation of proinflammatory regulatory networks centered around NF-kB, highlighting a potential mechanistic link to chronic immune dys-regulation during the post-acute phase (106).

**Figure 2:**
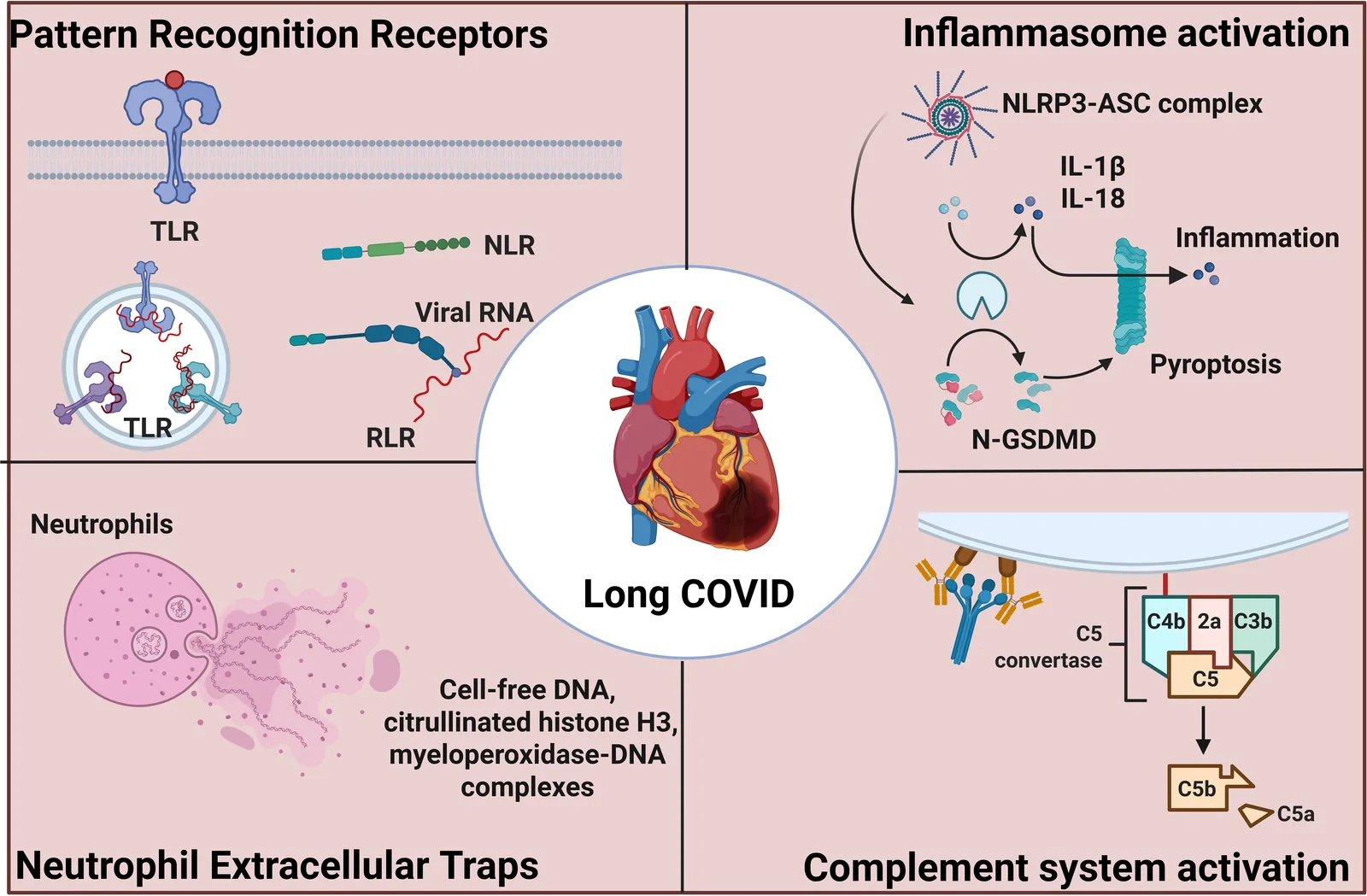
Innate immune dysregulation driving cardiac inflammation in Long COVID. Viral component sensing by PRRs triggers inflammasome activation and cytokine release, while neutrophil extracellular traps formation and complement system activation amplify inflammatory cascades. These interconnected mechanisms contribute to persistent inflammation and cardiac injury in Long COVID.

The vascular consequences of chronic PRR activation are multifold. Sustained NF-kB signaling in endothelial cells promotes expression of adhesion molecules such as ICAM-1, VCAM-1, and E-selectin, facilitating persistent recruitment of leukocytes into the vascular wall. These activated endothelial cells also secrete chemokines including CCL2 and CXCL8, further amplifying immune cell infiltration. Simultaneously, interferon signaling disrupts endothelial nitric oxide synthase (eNOS) activity and reduces nitric oxide bioavailability, impairing vasodilation and promoting vasoconstriction (107). Prolonged PRR activation also induces tissue factor and von Willebrand factor release, tipping the hemostatic balance toward thrombosis. The result is an endothelial phenotype that is adhesive, prothrombotic, and vasoconstrictive, creating fertile ground for microvascular occlusion and ischemic injury. Clinical studies support this mechanism. Convalescent COVID-19 patients with persistent cardiovascular symptoms exhibit elevated circulating soluble intercellular adhesion molecule 1 (ICAM-1) and vascular cell adhesion molecule 1 (VCAM-1), indicating endothelial activation. These patients also display increased levels of von Willebrand factor antigen, reflecting enhanced endothelial procoagulant activity (108). Together, these findings establish PRR hyper-activation as a critical driver of chronic endothelial dysfunction and cardiovascular injury in Long COVID (**Figure 2**).

### Neutrophil extracellular traps and immunothrombosis

Neutrophils represent another innate immune component profoundly dysregulated in Long COVID, with excessive and persistent formation of neutrophil extracellular traps (NETs) serving as a key mechanism of vascular injury and thrombosis. NETs are web-like structures composed of decondensed chromatin decorated with histones, neutrophil elastase, and myeloperoxidase, expelled by neutrophils during a specialized form of cell death termed NETosis (15). While NETs are effective at trapping pathogens, their persistence in circulation and tissues inflicts substantial collateral damage. In acute COVID-19, NETs are abundant in the lungs, circulation, and microvasculature, correlating with disease severity, respiratory failure, and mortality (109). Importantly, elevated markers of NETs persist in convalescent patients, including cell-free DNA, citrullinated histone H3, and myeloperoxidase-DNA complexes, indicating ongoing NETosis or defective clearance mechanisms (110). Notably, in Long COVID patients, persistent elevations of NET biomarkers are strongly associated with symptoms such as palpitations, tachycardia, and exercise intolerance, suggesting that NET-driven microvascular dysfunction underlies these clinical manifestations. Histopathological evidence supports this notion: cardiac and pulmonary tissue obtained from patients months after recovery reveals intravascular aggregates of neutrophils and extracellular chromatin consistent with NET remnants (111).

Mechanistically, NETs are profoundly deleterious to vascular homeostasis. Histones released in NETs are highly cytotoxic, inducing endothelial cell death and disrupting barrier function. Neutrophil elastase and myeloperoxidase degrade endothelial junctional proteins, further compromising vascular integrity (112). NET-derived DNA serves as a negatively charged scaffold that binds platelets and supports activation of the intrinsic coagulation cascade, while NET-associated tissue factor directly activates the extrinsic pathway. Together, these mechanisms drive excessive thrombin generation, fibrin deposition, and ultimately microvascular occlusion. Thus, NET dysregulation constitutes a central mechanism by which neutrophils contribute to cardiovascular sequelae in Long COVID (**Figure 2**).

### Complement pathway dysregulation and chronic vascular injury

Complement activation represents yet another axis of innate immune dys-regulation that persists in Long COVID and contributes to chronic cardiovascular injury. The complement system, composed of the classical, lectin, and alternative pathways, is designed to opsonize pathogens, recruit immune cells, and mediate lysis of infected targets. In acute COVID-19, uncontrolled complement activation is a hallmark of severe disease, with elevated C3a and C5a driving neutrophil and monocyte recruitment and the terminal complement complex (C5b-9) depositing on endothelial surfaces, leading to cell lysis and vascular injury (113). Beyond the acute infection, persistent complement activation has been reported in patients with Long COVID, suggesting incomplete resolution of innate immune responses. Longitudinal studies reveal persistent elevation of C3a, C5a, and soluble C5b-9 in convalescent patients, even in those without detectable viral replication (114). Consistent with these systemic findings, myocardial biopsies from convalescent patients demonstrate persistent deposition of C3 and C5b-9 in coronary microvasculature, correlating with clinical presentations of chest pain, arrhythmias, and reduced cardiac function (115). Mechanistically, sublytic C5b-9 deposition chronically activates endothelial cells, driving adhesion molecule expression and proinflammatory, prothrombotic signaling, while C5a-C5aR1 engagement amplifies neutrophils and monocytes recruitment and vascular inflammation. Complement also cross-activates coagulation cascades, with C5a inducing tissue factor expression and complement proteins binding fibrin and platelets to stabilize thrombi. These interactions ensure that complement dysregulation is intimately tied to thrombosis and vascular injury. Taken together, these findings underscore that complement is not merely an acute effector mechanism but a chronic amplifier of cardiovascular pathology in Long COVID (**Figure 2**).

### Inflammasome activation and pyroptotic injury

Alongside persistent PRR signaling, over-activated inflammasome pathway-particularly the NLRP3 inflammasome-represents another key mechanism linking innate immune activation to cardiovascular sequelae of Long COVID (116). The inflammasome is a multiprotein complex that serves as a cytosolic sensor of microbial products and sterile danger signals (117). Upon activation, NLRP3 recruits the adaptor ASC and pro-caspase-1, leading to auto-catalytic activation of pro-caspase-1 (118). Activated caspase-1 cleaves pro-IL-1b and pro-IL-18 into their mature forms and induces pyroptotic cell death through cleavage of gasdermin D (GSDMD) (119, 120). While transient inflammasome activation contributes to antiviral defense, its chronic engagement is deleterious, sustaining vascular inflammation and tissue injury (121).

**Figure 3:**
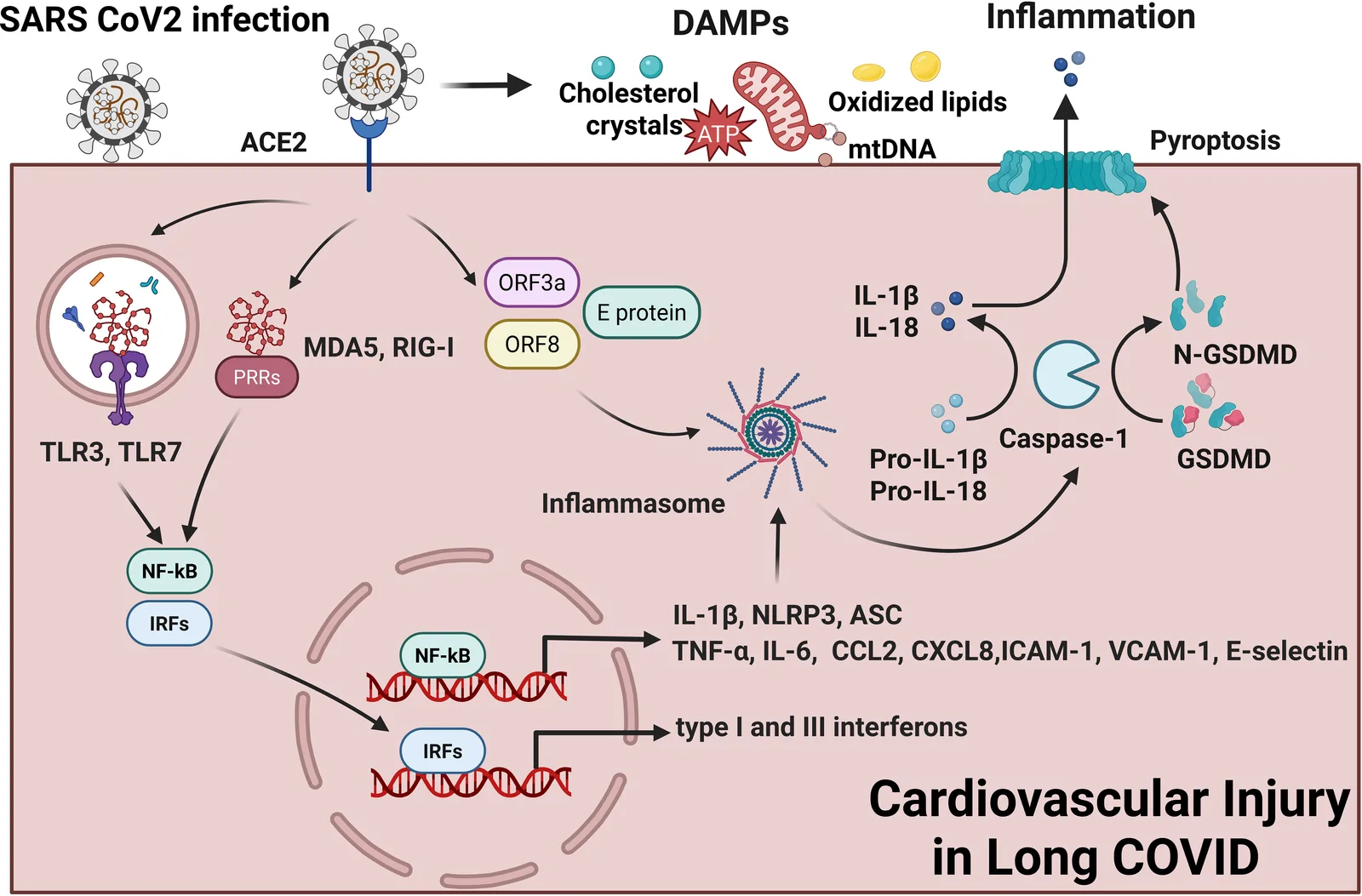
Recognition by PRRs and inflammasome activation in cardiovascular injury in Long COVID. Viral sensing by PRRs and the release of damage-associated molecular patterns (DAMPs) promote NF-kB signaling, interferon responses, and inflammasome assembly. This leads to caspase-1 activation, cytokines release (IL-1b, TNF-a, IL-6, CCL2, CXCL8, ICAM-1, VCAM-1, E-selectin, type I and III interferons), and gasdermin D (GSDMD)-mediated pyroptosis, resulting in persistent inflammation and cardiovascular injury.

During acute SARS-CoV-2 infection, several viral proteins directly activate the NLRP3 inflammasome. For instance, ORF3a functions as a viroporin, inducing potassium efflux and ROS generation, while ORF8 and the E protein disrupt organelle integrity, all of which promote NLRP3 activation (122). Beyond direct viral triggers, inflammasome activation persists in convalescent patients due to ongoing exposure to sterile DAMPs such as extracellular ATP, cholesterol crystals, mitochondrial DNA, and oxidized lipids released from damaged cells (123). These signals sustain caspase-1 activation in monocytes, macrophages, and vascular endothelial cells, long after viral clearance. Clinical studies demonstrate that IL-1b and IL-18 levels remain elevated in subsets of Long COVID patients, correlating with symptoms such as chest pain, exertional intolerance, and palpitations (124). In the myocardium, cardiac-resident macrophages chronically engaged in inflammasome signaling secrete IL-1b, which impairs cardiomyocyte contractility, alters calcium handling, and induces arrhythmogenic remodeling. IL-18, in turn, promotes IFN-g production and enhances Th1 polarization, creating a proinflammatory milieu that supports myocardial fibrosis (125). Animal models of viral myocarditis corroborate these observations, showing that sustained inflammasome activation accelerates adverse ventricular remodeling and leads to progressive heart failure (126). Histopathological studies in postmortem COVID-19 tissues reveal increased expression of NLRP3 and ASC specks in vascular and cardiac tissues months after infection, providing direct evidence of persistent inflammasome activation (127). Furthermore, inflammasome-driven pyroptosis has several implications for cardiovascular injury. Endothelial cells undergoing pyroptosis lose barrier integrity, release nuclear and cytosolic contents, and generate prothrombotic microparticles enriched in tissue factor (128). These microparticles circulate systemically, amplifying coagulation cascades and propagating vascular injury. Collectively, these findings indicate that NLRP3 inflammasome dysregulation is not only a transient feature of acute COVID-19 but also a long-term driver of cardiovascular sequelae through pyroptosis, cytokines release, and maladaptive tissue remodeling (**Figure 3**).

## Adaptive immune perturbations in Long COVID

**Figure 4:**
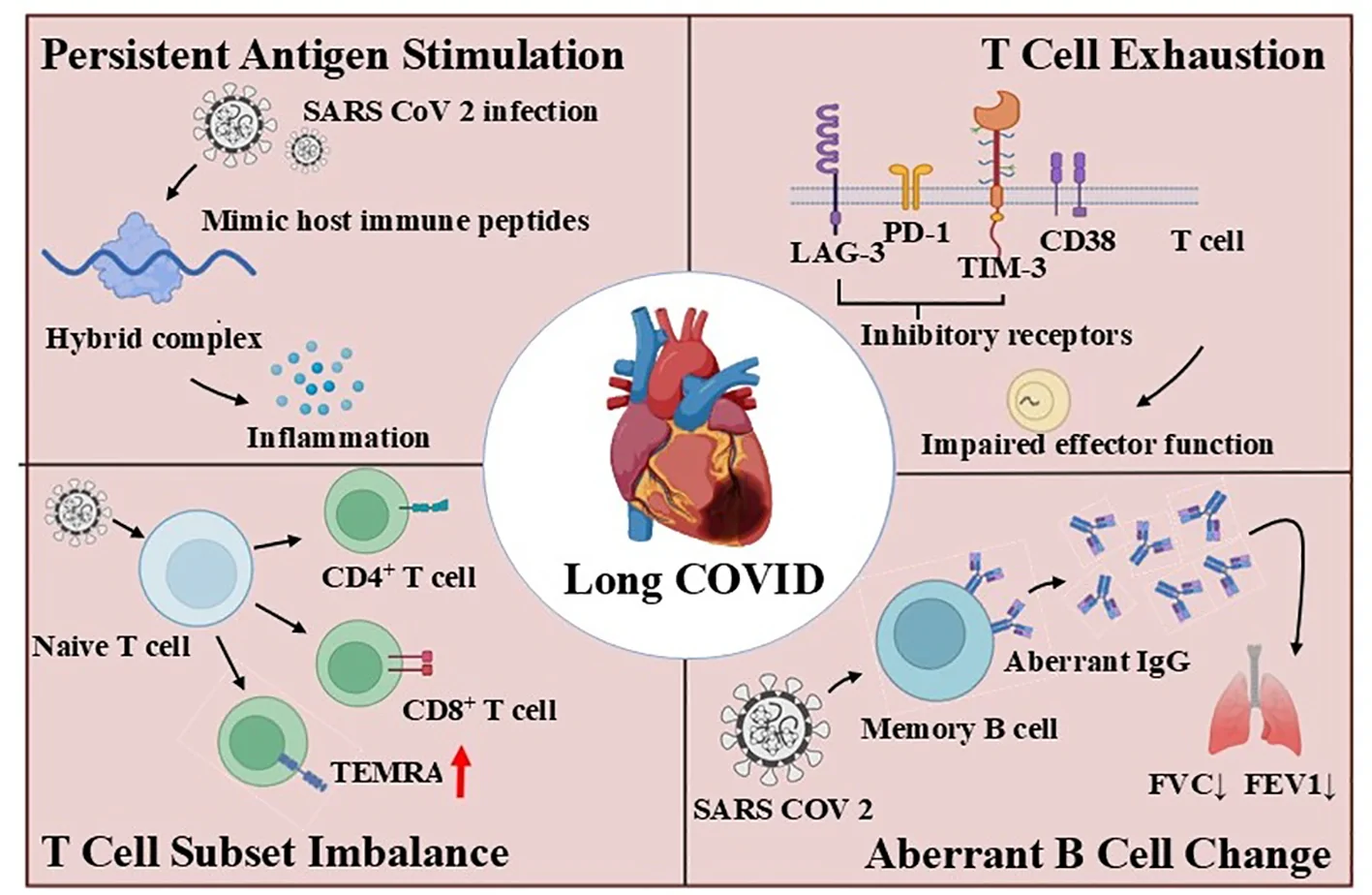
Integrated mechanisms of adaptive immune dysregulation in Long COVID. Persistent antigen stimulation following SARS-CoV-2 infection promotes molecular mimicry and the formation of hybrid immune complexes, leading to sustained inflammation. Chronic antigen exposure contributes to T cell exhaustion characterized by upregulation of inhibitory receptors, including PD-1, TIM-3, LAG-3, and CD38, resulting in impaired effector function. Additionally, T cell subset imbalance is observed, with naïve T cell depletion, altered CD4^+^ and CD8^+^ T cell responses, and expansion of terminally differentiated effector memory T cells re-expressing CD45RA (TEMRA). In parallel, aberrant B cell responses involving memory B cells and dysregulated immunoglobulin production contribute to abnormal IgG responses and are associated with reduced pulmonary function (FVC, FEV1).

Sustained alterations in adaptive immunity have also emerged as a defining feature of Long COVID. Growing evidence indicates that persistent antigen stimulation, T-cell exhaustion, imbalance of T-cell subsets, and dysregulated B-cell responses collectively contribute to chronic inflammation in affected individuals. Supporting this notion, patients with Long COVID exhibit increased frequencies of CD4^+^ T cells primed for migration to inflamed tissues, along with exhausted SARS-CoV-2 specific CD8^+^ T cells (**Figure 4**). These alterations are accompanied by elevated SARS-CoV-2–specific antibody levels and impaired coordination between virus-specific T- and B-cell responses, reflecting a mis-coordination of adaptive immune homeostasis (129).

### Persistent antigen stimulation

Residual SARS-CoV-2 antigens, or low-level viral reservoirs, may persist for months after acute infection and continually activate lymphocytes. This prolonged stimulation is a key factor in sustaining immune dysfunction during convalescence. Recent studies have demonstrated the presence of SARS-CoV-2 RNA fragments in Long COVID patients, suggesting active low-level viral replication. These viral fragments can mimic host immune peptides and form hybrid complexes that hijack immune response, leading to continuous immune activation and cytokine release (99). Additionally, elevated expression of viral entry-related genes and persistent host inflammatory transcripts in peripheral blood mononuclear cells (PBMCs) further support the notion of antigen persistence as a central mechanism (130).

### T cell exhaustion

Chronic immune activation has been associated with T cell exhaustion, a state defined by impaired effector function and sustained expression of inhibitory receptors such as PD-1, LAG-3, and TIM-3 (131). Multiple studies have reported the up-regulation of these markers in convalescent individuals with persistent symptoms. A cohort study tracking patients up to 18 months after SARS-CoV-2 infection found significantly elevated plasma levels of sCD25, sTIM-3, and sLAG-3 in individuals with Long COVID compared to healthy controls (131). These elevations were particularly prominent in patients experiencing persistent symptoms such as fatigue, memory impairment, and dyspnea. Notably, sCD25 levels showed a positive correlation with SARS-CoV-2-specific antibody titers, implying a potential feedback loop between humoral and cellular immune activation that may contribute to chronic immune dysfunction. In addition, exhausted CD38^+^ HLA-DR^+^ T cells also showed signs of enhanced MHC-II-mediated cross-talk and cytoskeletal remodeling, suggesting aberrant antigen presentation and prolonged synaptic interactions (132). These features may impair T cell contraction and memory formation, thereby prompting long-term immune dysfunction.

### T cell subset imbalance

SARS-CoV-2 infection disrupts the homeostasis of T cell subsets, a disturbance that can contribute to over-activated inflammation. Such persistent dysregulation of T cell composition has been closely linked to the pathogenesis of Long COVID. In a six-month follow-up cohort, patients with Long COVID showed elevated levels of CD45RA^+^ effector memory T cells (TEMRA) in response to viral nucleocapsid and membrane proteins, indicating repetitive antigenic stimulation and reduced memory recall capacity (133). Supporting this, a machine learning model identified skewing of T cell subsets as one of the most predictive features for persistent Long COVID symptoms, highlighting its potential clinical significance. Distinct immune cell phenotypes are associated with COVID-19 disease severity and symptom persistence. High-dimensional mass cytometry revealed that asymptomatic individuals exhibited higher frequencies of polyfunctional CD4^+^ and CD8^+^ T cells, as well as regulatory NK cell subsets (134). In contrast, symptomatic and Long COVID patients showed elevated levels of inflammatory cytokines alongside a reduction in regulatory immune populations, suggesting a shift toward a pro-inflammatory immune profile.

### Aberrant B cell change

In addition to sustained antigen stimulation and T cell response, Long COVID has increasingly been linked to aberrant B cell activation and altered subset distribution. Memory B cells appear to remain activated beyond the acute phase, producing high levels of SARS-CoV-2-specific IgG months after infection and vaccination (135). A longitudinal study further revealed that Long COVID patients exhibited significantly elevated receptor-binding domain (RBD)-specific IgG titers compared with healthy controls, particularly among those with moderate to severe initial disease. Notably, higher antibody titers were negatively correlated with pulmonary function tests such as FVC and FEV1, suggesting immune-mediated tissue injury (136). Beyond sustained antibody production, emerging evidence indicates that alterations in B cell subset distribution, alongside skewed cytokine responses, may contribute to the chronic inflammation. Immunophenotypic analyses have revealed marked shifts in B cell subsets despite normal total CD19^+^ counts. Specifically, naïve mature B cells (CD27^−^CD38^+^) were elevated, while transitional (CD27^−^CD38^+^) and double-negative (CD27^−^CD38^−^) B cells - subsets implicated in immune tolerance - were significantly reduced. Together, these findings indicate that an imbalance characterized by increased naïve mature B cells and reduced transitional B cells may drive sustained antigenic stimulation, low-quality antibody production, and chronic inflammation. This dysregulated B cell profile could underpin hallmark symptoms such as fatigue, cognitive impairment, and dysautonomia, highlighting transitional B cells as both a potential biomarker and a therapeutic target in Long COVID.

## Limitations

In this review, we present a comprehensive overview of current clinical and experimental evidence regarding the cardiovascular sequelae of Long COVID and the underlying immune mechanisms, including innate and adaptive immune dysfunction. Despite accumulating evidence demonstrating that patients with Long COVID face a potentially higher risk of cardiovascular sequelae, several methodological limitations of the studies included in this review should be acknowledged. The prevalence estimates of cardiovascular diseases are highly sensitive to study design, population selection, measure methodology, and case definition. For example, a large cohort study using the US Veterans Affairs database demonstrated that individuals with Long COVID exhibited a significantly increased 1-year risk of incident cardiovascular diseases, beyond 30 days after infection (6). This study provides important insights but has several methodological limitations. The study cohort was predominantly composed of older male veterans, thereby limiting the generalizability of the findings to the broader population. Additionally, potential imbalances of the control groups in baseline health status and healthcare utilization may lead to an overestimation of cardiovascular risk. Furthermore, this study was conducted before widespread vaccination and the emergence of the Omicron variant, indicating that the reported cardiovascular risks may not fully reflect the current clinical landscape, particularly in vaccinated populations or in the context of newer variants. Similarly, another study included in this review, which was also based on the US Veterans Affairs database, is subject to imbalances in study populations and variability in control group characteristics (88). It should also be noted that the use of non-standard CMR thresholds in the prospective cohort study cited in this review may have resulted in a considerably higher reported prevalence of myocarditis associated with Long COVID, which has not been consistently replicated in subsequent studies (43). Overall, these limitations highlight that current estimates of cardiovascular risk related to Long COVID should be interpreted with caution.

## Conclusion

Our understanding of the mechanisms and diagnostic criteria for Long COVID-associated cardiovascular injury remains incomplete. Therefore, identifying reliable diagnostic biomarkers and exploring novel therapeutic targets is urgently needed. Given the involvement of persistent innate and adaptive immune dysregulation, including sustained PRRs recognition, inflammasome activation, neutrophil extracellular trap formation, complement dysregulation, persistent antigen stimulation, T cell exhaustion, T cell subset imbalance, and dysregulated B cell responses in the progression of cardiovascular injury in Long COVID, the identification of these molecular signatures may provide clinically relevant biomarkers for diagnosis. In detail, persistent PRR activation drives the expression of NF-kB signaling in endothelial cells, leading to chronic endothelial dysfunction, microvascular obstruction, and long-term cardiovascular sequelae, including thrombosis and ischemic heart diseases, in Long COVID. Additionally, both neutrophil extracellular traps and complement dysregulation contribute to thrombus formation and chronic vascular injury. Sustained inflammasome activation is capable of promoting pathological ventricular remodeling, ultimately resulting in progressive HF.

Beyond the previously described immune dysregulation mechanisms underlying Long COVID-related cardiovascular diseases, recent studies have identified several novel mechanisms, including persistent activation of transient receptor potential cation channel subfamily M member 4 (TRPM4), noncanonical *in vivo* ferroptosis, and mitoxyperilysis. Noncanonical ferroptosis, an iron-dependent form of cell death distinct from apoptosis, is driven by reactive oxygen species (ROS)-induced peroxidation of phosphatidic acid (PA) (137). Necrocide 1 (NC1)-mediated persistent activation of TRPM4 (a nonselective cation channel) induces necrotic cell death characterized by sodium overload (138). Mitoxyperilysis, a newly described cellular death pathway, is characterized by mTORC2-mediated mitochondrial membrane lysis and cell death. This process is triggered by the synergy of innate immune activation and metabolic stress, with implications for inflammatory diseases and cancer therapy (139). These immune pathways are identified as the novel potential therapeutic targets for cancer or inflammatory diseases. However, it is unclear whether these immune pathways contribute to the pathogenesis of cardiovascular complications associated with Long COVID. Future research on these pathways may provide valuable insights for the development of targeted therapeutic strategies for cardiovascular sequelae associated with Long COVID.

In addition, accumulating evidence suggests that pre-existing comorbidities may increase susceptibility to cardiovascular sequelae associated with Long COVID, including acute myocardial infarction, heart failure, stroke, and thrombotic disease (140, 141). Individuals with underlying comorbidities, particularly diabetes, obesity, and cardiovascular disease (such as hypertension, coronary heart disease, and cardiomyopathy), commonly exhibit persistent inflammation, endothelial dysfunction, renin-angiotensin system dysregulation, and immune dysregulation, all of which may contribute to an increased risk of cardiovascular diseases in Long COVID (141, 142). Notably, the interplay of pre-existing comorbidities and cardiovascular sequelae of Long COVID is complex and bidirectional (143). Long COVID patients with multimorbidity are at increased risk of cardiovascular sequelae and adverse clinical outcomes. At the same time, Long COVID can also aggravate the progression and severity of underlying chronic conditions. Considering the substantial interindividual heterogeneity in the development of cardiovascular sequelae, individualized cardiovascular surveillance and risk stratification strategies are essential for Long COVID patients with underlying comorbidities.

Finally, substantial heterogeneity has been observed in both the prevalence of cardiovascular sequelae and the expression of immune dysfunction among studies of Long COVID. Distinct immunological characteristics, such as persistent immune activation and impaired immune function, may contribute to differential susceptibility to cardiovascular complications. This heterogeneity highlights the potential value of personalized medicine approaches to guide immune-targeted interventions based on individual immunological profiles, which may ultimately improve cardiovascular outcomes, enhance quality of life, and reduce long-term healthcare burden in Long COVID patients.
