# Correction: Cardiovascular sequelae of Long COVID: immune dysregulation inflammation as central drivers

**DOI:** 10.3389/fimmu.2026.2025243

**Published:** 2026-10-29

**Authors:** Anhui Liu, Hanbing Chen, Qingxiang Liu, Mahmoud Al-Azab, Fahmi Shaher, Jiang Li, Tao Liu, Min Yang

**Affiliations:** 1 Wuxi School of Medicine, Jiangnan University, Wuxi, China; 2 Department of Cardiac Vascular Surgery, Affiliated Hospital of Jiangnan University, Wuxi, China; 3 Department of Cardiac Surgery, Peking University People’s Hospital, Peking University, Beijing, China; 4 School of Medicine, Southeast University, Nanjing, China; 5 Department of Emergency Medicine, The First Affiliated Hospital with Nanjing Medical University, Nanjing, China; 6 School of Basic Medical Sciences, Guangzhou Women and Children’s Medical Centre, Guangzhou Medical University, Guangzhou, China; 7 Department of Medical Microbiology, Faculty of Medicine, University of Science and Technology, Aden, Yemen; 8 Department of Basic Sciences, Faculty of Medicine, University of Science and Technology, Aden, Yemen; 9 Clinical Big Data Research Center, The Seventh Affiliated Hospital of Sun Yat-sen University, Shenzhen, China; 10 State Key Laboratory of Respiratory Disease, National Clinical Research Center for Respiratory Disease, National Center for Respiratory Medicine, Joint International Research Laboratory of Respiratory Health, Guangdong Basic Research Center of Excellence for Respiratory Medicine, Department of Allergy and Clinical Immunology, Guangzhou Institute of Respiratory Health, The First Affiliated Hospital of Guangzhou Medical University, Guangzhou, China

**Keywords:** adaptive immune response, cardiovascular sequelae, immune dysregulation, innate immune response, Long COVID

The reference for 136 was erroneously written as “Massard M et al. Modelling and investigating memory immune responses in infectious disease. Application to influenza a virus and sars-cov-2 reinfections. Infect Dis Model (2025) 10:163–188. doi: 10.1016/j.idm.2024.09.009”. It should be “Siddik AB et al. Pulmonary function and comparative SARS-CoV-2 RBD-specific IgG antibody response among the COVID-19 recovered group. PLOS One (2025) 20:e0318959. doi: 10.1371/journal.pone.0318959”.

The original version of this article has been updated.

